# Delayed diagnosis of thoracic esophageal rupture due to blunt abdominal trauma without chest trauma: A case report

**DOI:** 10.1186/s13019-022-01971-y

**Published:** 2022-09-03

**Authors:** Jae Jun Kim, Jung Wook Han

**Affiliations:** Department of Thoracic and Cardiovascular Surgery, College of Medicine, Uijeongbu St. Mary’s Hospital, The Catholic University of Korea, 271 Cheonbo-ro, Uijeongbu-si, Gyeonggi-do Republic of Korea

**Keywords:** Thoracic esophageal rupture, Blunt abdominal trauma, Esophageal stent

## Abstract

**Background:**

Thoracic esophageal rupture due to blunt trauma is very rare. Moreover, there have been no reports of thoracic esophageal rupture due to blunt abdominal trauma without chest trauma.

**Case presentation:**

We describe a rare case of esophageal rupture due to blunt abdominal trauma in a young female patient. Operation was delayed due to a misdiagnosis of chylothorax, and esophageal repair was performed six days after trauma. Postoperative esophageal leak developed and was treated with esophageal stent. She was discharged two months after surgery without sequelae.

**Conclusions:**

It is important to consider esophageal rupture as a differential diagnosis even in patients with only abdominal trauma, when in doubt.

## Background

Thoracic esophageal rupture due to blunt trauma is an extremely rare clinical condition associated with significant morbidity and mortality. It is mostly caused by blunt trauma to the chest or to the chest and abdomen. There have been no reports of thoracic esophageal rupture due to blunt abdominal trauma without chest trauma. Here, we report a case of delayed diagnosis of thoracic esophageal rupture due to blunt abdominal trauma, treated with esophageal stent after primary repair.

## Case presentation

**Fig. 1:**
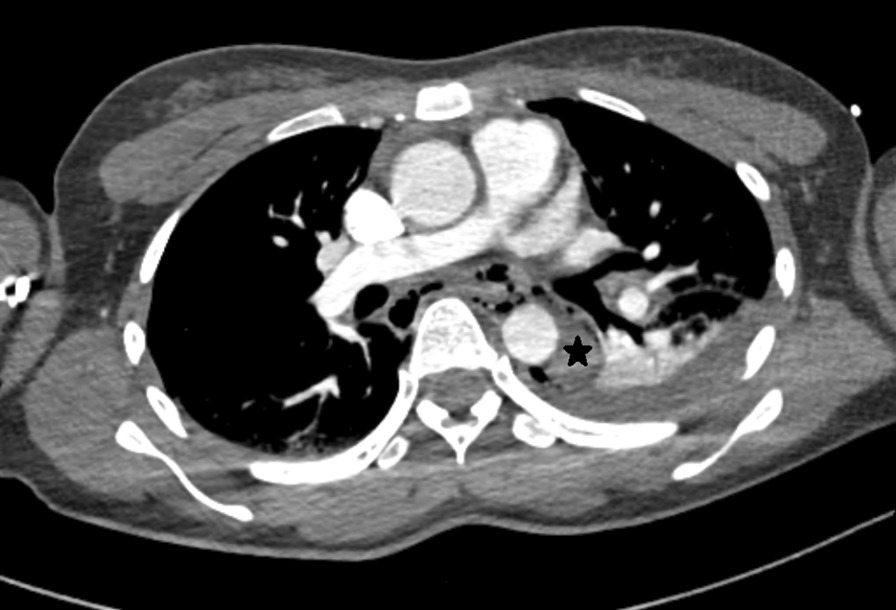
Chest CT on admission revealing abnormal fluid collection surrounding descending thoracic aorta (*)

A 29-year-old female patient was referred to our hospital due to abdominal crush injury by a reverse vehicle. She complained of dyspnea and abdominal pain. On admission, she was conscious. Her vital signs were as follows: blood pressure of 102/60 mmHg, heart rate of 135 beats/min, respiration rate of 20 breaths/min, and temperature of 38 °C. Focused assessment with sonography in trauma (FAST) was positive in the abdomen and left thorax. Chest computed tomography (CT) revealed pneumomediastinum, left lower lobe atelectasis, left hydrothorax and fluid collection in posterior mediastinum, surrounding mid to distal esophagus, and descending thoracic aorta without abnormality in bony thorax (Fig. 1). Abdomen and pelvic CT revealed liver laceration (segment 2–4, Gr III) and both superior and inferior pelvic rami fractures. A 24 French chest tube was inserted onto the left thorax by a trauma surgeon. Initially, her condition was considered as chylothorax because the drained pleural fluid was milky and there was no definitive chest trauma. She underwent emergency transcatheter arterial embolization for liver laceration. She was admitted to trauma intensive care unit by the Department of Trauma Surgery.

**Fig. 2:**
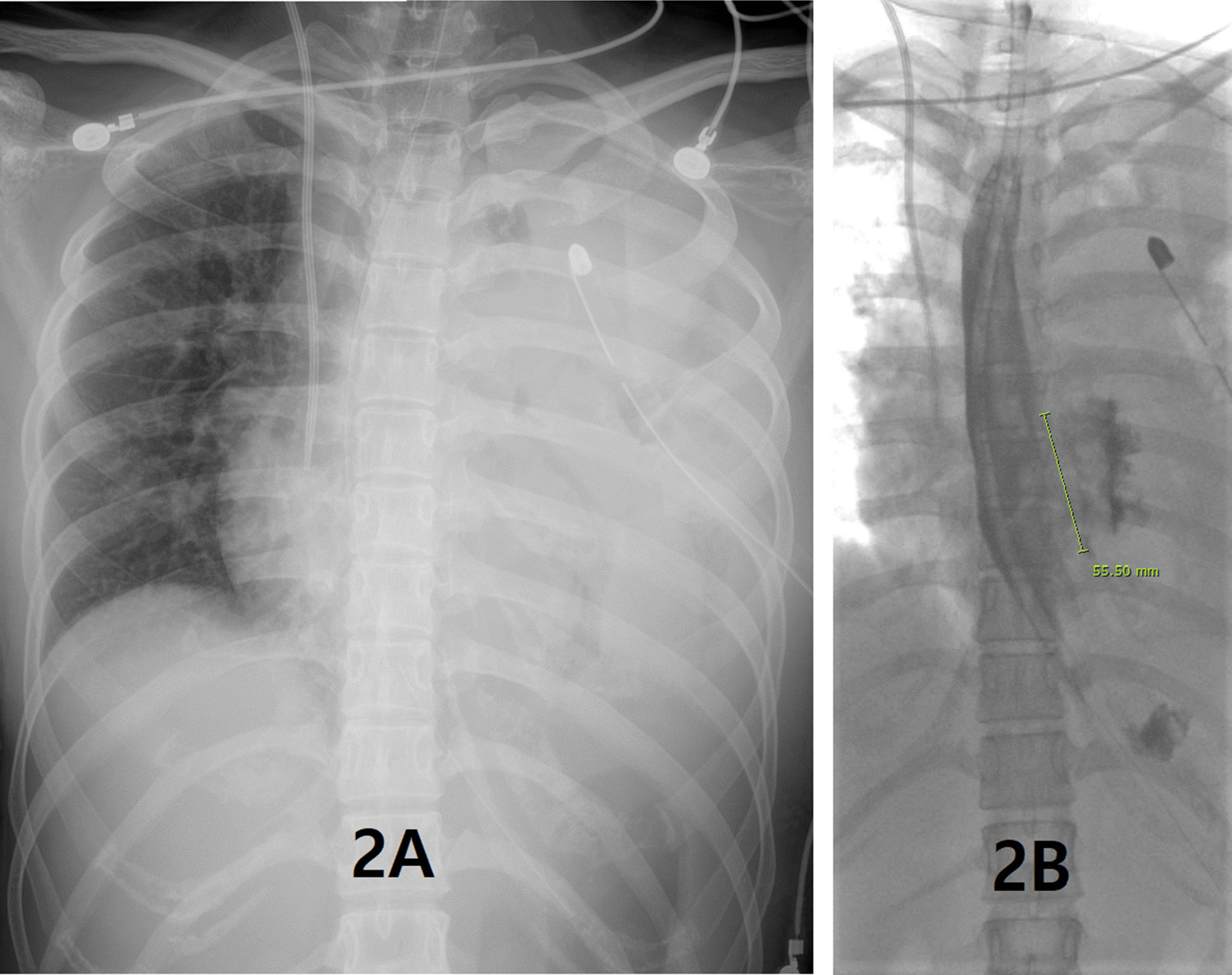
**A** Chest X ray revealing worsening of left lung field haziness. **B** Esophagography revealing about 5.5 cm in length contrast leakage from mid to lower thoracic esophagus

**Fig. 3:**
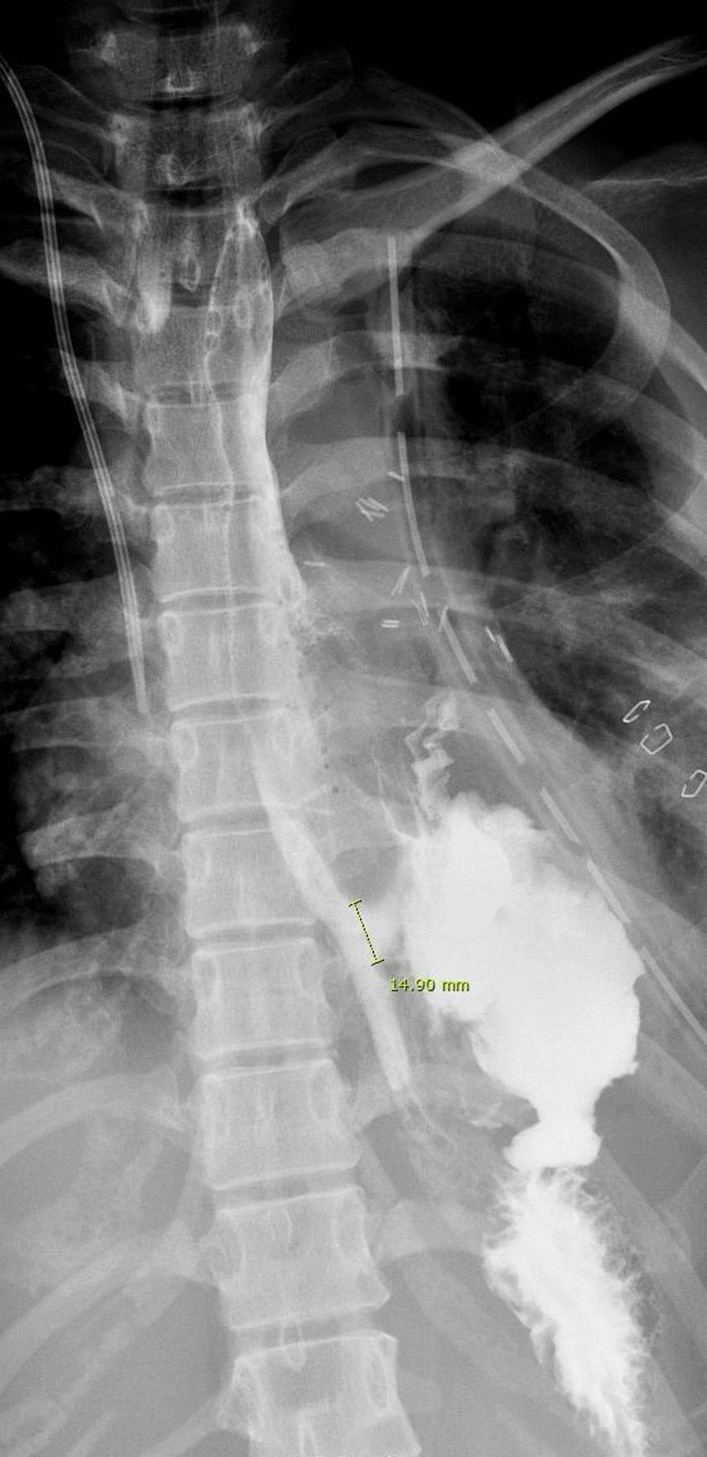
Esophagography on the 8th day postoperatively revealing about 1.5 cm length contrast leakage from distal thoracic esophagus

**Fig. 4:**
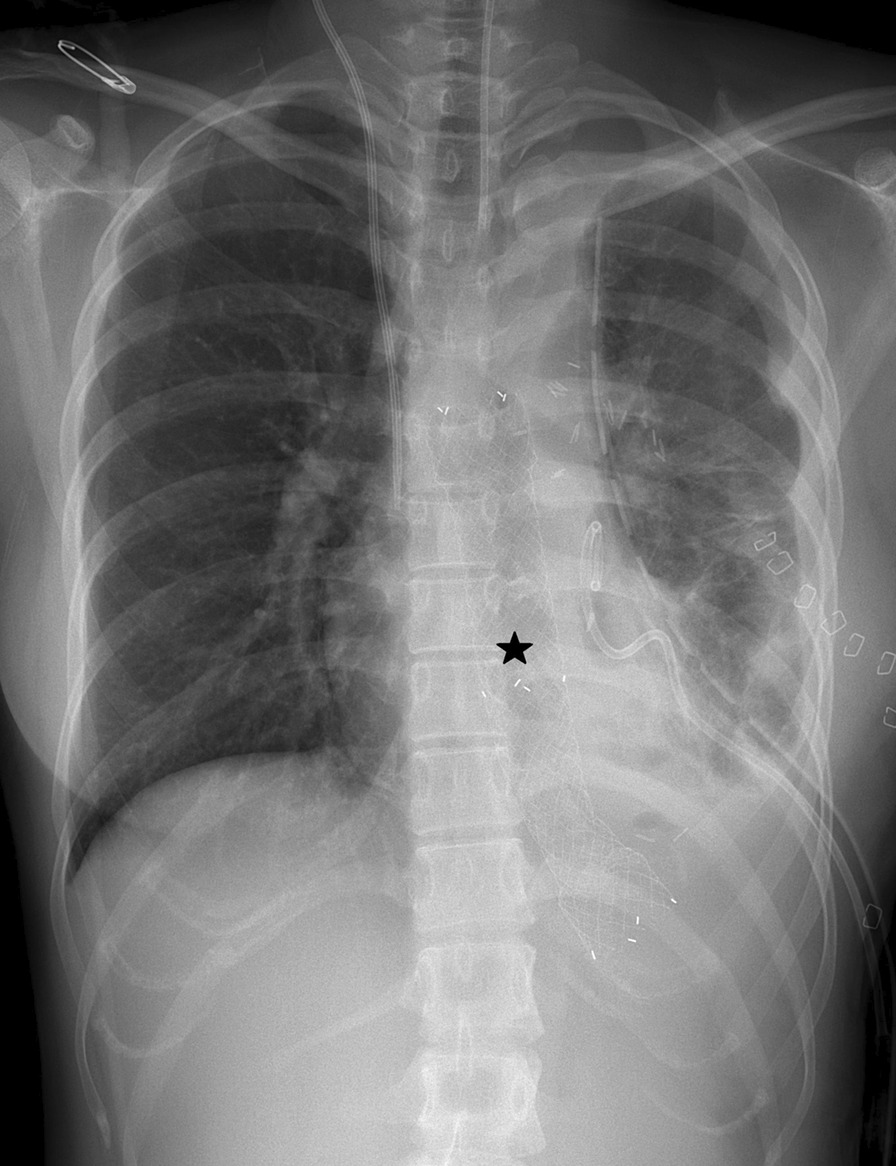
Esophageal stent observed on chest X-ray (*)

She was referred to the Department of Thoracic and Cardiovascular Surgery on the 5th day of hospitalization for worsening of left lung field haziness and her condition (Fig. 2A). Thoracic esophageal injury was suspected. Esophagography revealed mid to distal thoracic esophageal rupture (Fig. 2B). Emergency operation was performed by posterolateral thoracotomy through the left 6th intercostal space. On operative findings, multiple loculated effusion and longitudinal tear of mid to lower thoracic esophagus of about 8 cm in length were identified. After debridement of infected tissue and massive saline irrigation, primary repair was performed with interrupted sutures using 3–0 Vicryl. The chest was closed with a 24 French chest tube drainage. Esophagography at 8 days after operation revealed postoperative esophageal leak (Fig. 3), and esophageal stent placement was performed (Fig. 4). Postoperative esophageal leak was no longer observed on follow-up esophagography. The esophageal stent was removed 6 weeks after the placement. She was discharged two months after surgery. She was closely followed up without complications.

## Discussion

The incidence of esophageal rupture due to blunt chest trauma has been reported to be 0.001% [1]. Its mortality rate was about 20% in a recently published large case series [2]. Mortality rate is increased when there is a delay in its diagnosis. Some papers report that if treatment is delayed for more than 24 h after injury, mortality could increase by nearly 50% [3]. Unfortunately, 70% of esophageal rupture due to blunt trauma is diagnosed late with a poor prognosis [4].

The treatment strategy for esophageal rupture has not been established yet. Several studies have shown that early diagnosis and management are associated with good clinical outcomes and that the golden time for treatment is usually within 24 h [5]. Whether to perform primary repair or not depends on time. If esophageal rupture is diagnosed within 24 h of injury and the patient is stable, primary repair with or without pleural or muscle flap may be attempted [5]. However, if more than 24 h have passed since injury and if a patient is unstable, other methods such as esophageal diversion and exclusion should be considered [5].

Indications for esophageal stent are usually divided into two groups. The first indication is malignant or benign dysphagia [6]. The second indication is esophageal leakage [6]. Although there is no absolute contraindication, esophageal perforation longer than 6 cm is a relative contraindication [7]. The advantage of esophageal stent is that it can provide nutritional support through early feeding. However, a relatively high risk of adverse events, particularly stent migration, is a major limitation of the use of esophageal stent [6].

In our case, we missed the possibility of thoracic esophageal rupture because there was liver laceration required emergent treatment and chylothorax can develop after abdominal trauma without chest trauma. The patient’s condition was getting worse and total left lung field haziness led to surgery on the sixth day after trauma. According to the operative findings, most of esophageal mucosa was intact, primary repair was performed. However postoperative esophageal leak occurred due to delay in diagnosis and surgery. Considering the patient’s poor condition, future quality of life and the relatively small size of esophageal leak, redo esophageal repair or other surgery, such as esophageal exclusion and diversion, was inappropriate. So, we decided to treat postoperative esophageal leak with esophageal stent. Esophageal stent was maintained for six weeks without complications. No leaks or other complications remained after stent removal.

## Conclusions

This case report shows that, although rare, thoracic esophageal rupture can occur with only blunt abdominal trauma. Therefore, it is important to consider esophageal rupture as a differential diagnosis even in patients with only abdominal trauma, when in doubt.

## Data Availability

The datasets of the current study are available from the corresponding author upon reasonable request.

## Abbreviation

CT: Computed tomography
